# *Chlamydia pecorum* detection in aborted and stillborn lambs from Western Australia

**DOI:** 10.1186/s13567-021-00950-w

**Published:** 2021-06-11

**Authors:** Tom Clune, Shane Besier, Sam Hair, Serina Hancock, Amy Lockwood, Andrew Thompson, Martina Jelocnik, Caroline Jacobson

**Affiliations:** 1 Centre for Animal Production and Health, Murdoch University, South Street, Murdoch, WA 6150 Australia; 2 Department of Primary Industries and Regional Development, South Perth, WA 6151 Australia; 3 Genecology Research Centre, University of the Sunshine Coast, 90 Sippy Downs Drive, Sippy Downs, QLD 4557 Australia

**Keywords:** Chlamydia, Sheep, Reproduction, Abortion, Stillbirth, Dystocia, Lamb survival, Necropsy

## Abstract

Lamb survival is an important welfare and productivity issue for sheep industries worldwide. Lower lamb survival has been reported for primiparous ewes, but the causes of this are not well studied. The aim of this study was to determine causes of perinatal deaths for lambs born to primiparous ewes in Western Australia, and identify if infectious diseases are implicated. Lamb mortality from birth to marking were determined for 11 primiparous ewe flocks on 10 farms in Western Australia. Lamb mortality from birth to marking averaged 14% for single-born and 26% for multiple-born lambs. Lamb necropsies (*n* = 298) identified starvation–mismosthering–exposure (34%), dystocia (24%) and stillbirth (15%) as the most common causes of perinatal lamb death. There was no evidence of exotic abortigenic pathogens in aborted and stillborn lambs (*n* = 35). *Chlamydia pecorum* was detected by qPCR in 15/35 aborted and stillborn lambs on 5/6 farms. Preliminary molecular characterisation of *C. pecorum* detected in samples from aborted and stillborn lambs (*n* = 8) using both Multilocus Sequence Typing and *omp*A genotyping indicated all strains were genetically identical to previously described pathogenic livestock strains, denoted ST23, and dissimilar to gastrointestinal strains. High frequency of detection of a pathogenic *C. pecorum* strains ST23 associated with ovine abortion and stillbirth on multiple farms located across a wide geographic area has not been previously reported. *Chlamydia pecorum* may contribute to reproductive wastage for primiparous sheep in Western Australia. Further investigation to understand *C. pecorum* epidemiology and impact on sheep reproduction is warranted.

**Supplementary Information:**

The online version contains supplementary material available at 10.1186/s13567-021-00950-w.

## Introduction

Improving lamb survival is an important economic and welfare issue for sheep industries worldwide. Approximately 10% of single-born lambs and 30% of twin-born lambs die prior to weaning under extensive grazing conditions across Australia, with most losses occurring in the first 48 h of life [1–3]. The starvation–mismosthering–exposure complex, stillbirths, and dystocia are the most common causes of lamb mortality during the perinatal period [1, 2, 4]. Lower lamb survival has been reported for primiparous ewes compared to adult flocks in Australia [5–7] and overseas [6, 8–11]. However, causes of mortality for lambs born to primiparous ewes are not well described and it is not clear if the main factors contributing to lamb mortality in primiparous ewes are similar to those for multiparous ewes.

Infectious diseases may contribute to lamb mortality through abortion, stillbirths and birth of weak lambs that are more likely to die soon after birth. Campylobacteriosis, listeriosis and toxoplasmosis were the most common aetiological agents identified in sheep abortion investigations submitted to Australian veterinary diagnostic laboratories between 2006 and 2019 [12, 13]. This is consistent with older reports describing these as the most common infectious causes of abortion and perinatal mortality in Australian sheep [14–17]. Sporadic abortion associated with *Chlamydia pecorum* has been reported in sheep from Australia [12, 18] and overseas [19, 20], but the epidemiology of *C. pecorum*-associated abortion in sheep remains poorly understood.

Primiparous ewes may be more susceptible to infectious diseases, as younger ewes are less likely to have developed immunocompetency to infection prior to pregnancy [21, 22]. Most recent Australian studies that included lamb necropsies were conducted with multiparous ewes, and cause of death was assigned based on gross post-mortem findings without adjunct laboratory investigation [2, 23–27]. In general, relatively few investigations for abortion and perinatal lamb death are submitted to veterinary diagnostic laboratories for exclusion of infectious diseases [12]. Consequently, the role of infectious diseases as a contributor to mortality of lambs born to primiparous ewes are not well described and it is possible that the contribution of infectious disease to perinatal mortality may be underrecognized.

The aim of this study was to determine the common causes of perinatal death for lambs born to primiparous ewes, and whether infectious disease was implicated. In doing so, we identified *C. pecorum* in a surprisingly high proportion of aborted and stillborn lambs from multiple farms, and subsequently expanded the study to determine molecular characteristics for *C. pecorum* strains detected in aborted and stillborn lambs.

## Materials and methods

### Animals and study sites

Eleven flocks of primiparous ewes from ten farms were monitored between the start of mating and lamb marking at approximately 6 weeks from the start of lambing (Table 1). All farms were located in southern Western Australia in a region with Mediterranean climate characterised by hot dry summer and cool wet winter.

**Table 1:** **Characteristics of primiparous ewe flocks from Western Australia**

Flock code	Year	Location	Ewe breed	Ewe age at mating (months)	Pregnant ewes (*n*)^a^	Pre-lambing body condition score^b^
A	2018	Kojonup	Merino	18–20	186	2.7
B	2018	Kojonup	Merino	18–20	178	2.7
C	2019	Katanning	Merino	18–20	204	2.8
D	2019	Broomehill	Merino	18–20	169	2.5
E	2019	Katanning	Merino	7–9	86	2.7
F1	2018	Narrogin	Composite^c^	7–9	148	3.1
F2	2019	Narrogin	Composite^c^	7–9	168	3.1
G	2018	York	Composite^c^ & White Suffolk	7–9	130	3.1
H	2019	Kojonup	Composite^c^	7–9	151	3
I	2019	Kojonup	Dorper	8–10	146	3
J	2019	Ongerup	White Suffolk	7–10	103	3.1

^a^Determined by transabdominal ultrasound conducted 62–87 days from the start of mating or artificial insemination.

^b^Average body condition score of the flock assessed approximately 140 days from the start of mating.

^c^Composite: mixed (non-Merino) breed ewes.

On each farm, Merino or non-Merino ewes were mated as either ewe lambs (7–10 months, *n* = 7 flocks) or primiparous yearling ewes (18–20 months, *n* = 4 flocks; Table 1). All rams were of the same breed as the ewes to which they were joined. Rams were confirmed seronegative for *Brucella ovis* prior to mating using a modified complement fixation test [28] where three local *B. ovis* isolates were used as the antigen (DDLS freeze-dried culture collection numbers 0735, 1655, 1794). On two farms, ewes were artificially inseminated followed by a period of natural mating. All other flocks were mated naturally with an average mating period of 38 days (range 32–46 days). All ewes were managed as per standard farm practice including use of body condition monitoring to guide nutrition and grazing management, with no experimental interventions imposed by this study other than monitoring of ewes and lambs as described (Table 1).

### Measurements

Ewes were pregnancy scanned via transabdominal ultrasonography at 62–87 days from the start of mating to determine litter size and foetal viability. Ewe body condition score was recorded at approximately 140 days from the start of mating using a scale of 1 (very thin) to 5 (very fat) as previously described [29].

Farm staff checked the lambing flocks once or twice daily throughout the lambing period. Lambs were identified with an ear tag and their birth type and dam pedigree were recorded within 24 h of birth for most (8/11) flocks. The total number of lambs born for each flock was calculated using records of the number of lambs tagged at birth plus the number of dead lambs collected. On farms where tagging at birth was not performed, the number of lambs born was calculated based on number of lambs present at marking plus the number of dead lambs collected. Number of lambs born may have been underestimated at these sites because it is unlikely that all lambs that died were recovered for necropsy.

### Lamb necropsies and sample collection

Lambs that died in the first three days following birth were retained for necropsy to determine cause of death. Dead lambs were either refrigerated (4 °C) or frozen (−20 °C) for up to 5 days before necropsies were performed. One aborted foetus and one foetal membrane were also recovered from Flock F1 prior to the start of lambing and submitted for necropsy and diagnostic testing for infectious agents.

Lamb necropsies were performed by a single person using methods described by Everett-Hincks and Duncan [30]. Briefly, post-mortem examination included recording the weight, sex and details of the external appearance of the lamb along with gross examination of thoracic and abdominal organs. Brain tissues were assessed for lesions only in lambs that had not been frozen. Cause of death was classified according to methods previously described [30], and described in more detail in Additional file 1.

Tissue samples from aborted or stillborn lambs from flocks with at least two lambs classified as abortion or stillbirth were submitted to the Department of Primary Industry and Regional Development Diagnostic Laboratory Service (South Perth, Western Australia). The type of tissues submitted varied between cases (Additional file 2), with liver and placenta submitted for all cases except where these were not available due to predation.

### Laboratory investigation

Histology, bacteriology and molecular diagnostics for endemic and exotic abortigenic agents were performed by the Department of Primary Industry and Regional Development’s Diagnostics and Laboratory Services. Bacteriology comprised culture on blood agar plus selective culture for *Salmonella* spp., *Campylobacter* spp. and *Listeria* spp.. Molecular testing included polymerase chain reactions (PCRs) for *Brucella* spp., *Campylobacter* spp., *Leptospira* spp., *Toxoplasma gondii*, *Coxiella burnetii*, pestiviruses and *Chlamydia* spp. that are described in more detail below.

All aborted tissue samples underwent routine bacteriological culture on Columbia agar (Oxoid) with 5% equine blood and MacConkey agar (Oxoid). Additionally, foetal liver, cotyledon and placenta samples were subject to selective isolation for *Listeria, Salmonella* and *Campylobacter* (PathWest Media, Western Australia). All cultures were incubated at 37 °C with 5% CO_2_ except for the *Campylobacter* cultures which were incubated under microaerophilic conditions.

Histopathology was performed on formalin fixed tissues processed to haematoxylin and eosin (H&E) slides; a subset of cases was also subjected to immunohistochemistry. Representative specimens were processed from 10% buffered formalin solution to paraffin embedded tissue in a Logos Milestone histological processer and blocked using standard histological techniques. Sections were trimmed at 4 μm thickness and stained to H&E in a Leica autostainer XL with Leica CV5030 coverslipper. Selected sections were subject to immunohistochemistry with an anti-*Chlamydia* polyclonal antibody (B47829R, Progen) and an anti-*Toxoplasma gondii* polyclonal antibody (B65201R, Biodesign). Both antibodies were visualised using the Dako Envision Dual-link system and Dakocytomation DAB+ (both Dako, Agilent) according to the manufacturers’ instructions.

### Molecular testing—nucleic acid extraction

DNA extraction was performed using the QIAamp DNA Mini Kit (Qiagen) and run on the automated Qiacube platform (Qiagen) following the Purification of DNA from tissues protocol. RNA was extracted using the MagMAX-96 Viral RNA Isolation Kit (Thermo Fisher Scientific) on the MagMAX Express-96 (Thermo Fisher Scientific) magnetic bead processor.

### Molecular testing—*Chlamydia* spp.

*Chlamydia* quantitative polymerase chain reactions (qPCRs), targeting the outer membrane protein A (*omp*A) gene, were performed on all foetal liver, cotyledon and placenta samples using species-specific assays for the detection of *C. pecorum* [31], *C. abortus* and *C. psittaci* [32]. Positive *C. pecorum* detections were confirmed via 298 bp and 806 bp *Chlamydiales* 16S rRNA gene fragments PCRs [33] followed by Sanger sequencing.

*Chlamydia* qPCR assays were performed in 25 µL reaction volumes containing 0.5 µM of each primer, 0.2 µM of probe, 12.5 µL of Rotor-Gene Multiplex Master Mix (Qiagen) and 5 µL of extracted DNA. The qPCR reactions were run on a Rotor-Gene Q (Qiagen) real-time PCR cycler under the following conditions: initial denaturation at 95 °C for 5 min followed by 45 cycles of 95 °C for 15 s and 60 °C for 15 s with fluorescent probe acquisition occurring during the 60 °C annealing/extension step. A 25 µL reaction volume was also used for the *Chlamydiales* PCRs and contained 0.4 µM of each primer, 12.5 µL of HotStarTaq Master Mix (Qiagen) and 5 µL of extracted DNA. Conventional PCR was performed on a DNA Engine (Bio-Rad) thermal cycler under the following conditions: initial denaturation at 95 °C for 5 min followed by 40 cycles of 94 °C for 30 s, 55 °C for 45 s and 72 °C for 45 s with a final elongation step of 72 °C for 7 min. To minimise contamination risk synthetic positive control gBlocks Gene Fragments (Integrated DNA technologies) were designed for all PCRs.

Conventional PCR amplicons were purified using the Qiaquick PCR purification kit (Qiagen) and forward and reverse sequencing reactions were prepared in 12 μL volumes containing approximately 12–18 ng of PCR product and 9.6 pmol of primer. All amplicons were sequenced at the Australian Genome Research Facility (AGRF Perth Node) and sequence and BLAST analysis was performed using Geneious R11 [34].

### Molecular testing—other abortifacents

Screening for *Brucella* spp. [35], *Campylobacter* spp. [36], *Coxiella burnetii* [37, 38], pathogenic *Leptospira* spp. [39], *Toxoplasma gondii* (VetMAX *T. gondii* Kit—Thermo Fisher Scientific) and Pestivirus [40, 41] was undertaken via PCR. Further screening for *Brucella* spp. [42] was performed at The Australian Centre for Disease Preparedness, Geelong, Victoria. All primer sequences, probes, final concentrations, and cycling conditions for diagnostic PCRs and molecular testing performed in this study are outlined in Additional file 3.

### *C. pecorum* genotyping by Multi Locus Sequence Typing (MLST) and ompA

Prior to genotyping, the *C. pecorum* positive DNA samples from six liver and two cotyledon samples taken from eight aborted and/or stillborn lambs from four different farms (Additional file 2) were quantified for *C. pecorum* genome copy number (tested in duplicate) using standard curve calibrated and High Resolution Melt (HRM) *C. pecorum* qPCR assay [43]. The genome copy number/µL in samples ranged from 3.41 × 10^2^–2.47 × 10^5^ copies/µL DNA template, with geometric mean of 7.26 × 10^3^ copies/µL (Table 2). The *C. pecorum*-specific MLST [44, 45] and *ompA* gene sequence analyses [46] are the most commonly used molecular typing targets for *C. pecorum*, due to their recognised congruence with whole-genome phylogeny. Full-length *omp*A genotyping [47] and *C. pecorum* MLST [45] were applied as previously described to these eight *C. pecorum* positive DNA samples.

**Table 2:** **Mean** ***C. pecorum*** **loads detected by qPCR, and sequence type and sequence identity identified using MLST and ompA**

Strain name	Tissue	Mean Ct	Mean qPCR Loads (copies/uL)	MLST	ompA % sequence identity
FarmF1_Foetus1	Cotyledon	21.53	10 552	ST 23	100% E58
FarmF1_StillbornLamb2	Liver	21.24	12 900	ST 23	100% E58
FarmF1_Foetus3	Liver	18.46	88 353	ST 23	100% E58
FarmF1_StillbornLamb4	Liver	24.69	1176	ST 23	100% E58
FarmA_StillbornLamb1	Liver	26.47	341	ST 23	100% E58
FarmH_StillbornLamb1	Liver	22.09	7110	ST 23	100% E58
FarmH_StillbornLamb2	Cotyledon	16.97	247 296	ST 23	100% E58
FarmJ_StillbornLamb	Liver	25.06	911	ST 23	100% E58

The resultant MLST sequences were confirmed for sequence type (ST) by using the online *Chlamydiales* PubMLST database [48]. Both concatenated *C. pecorum* MLST, and *omp*A sequence and phylogenetic analyses were performed in GeneiousPrime 2020 [34]. The concatenated MLST sequences of eight samples from this study were aligned using ClustalOmega (as implemented in Geneious) to other 36 publicly available livestock *C. pecorum* MLST sequences retrieved from the *Chlamydiales* PubMLST database [48]. Using the concatenated MLST sequences 3095 bp alignment for the 44 *C. pecorum* global and Australian livestock strains, we have constructed a mid-point rooted approximately-maximum-likelihood phylogenetic tree, using FastTree 2.1.11 [49].

**Figure 1:**
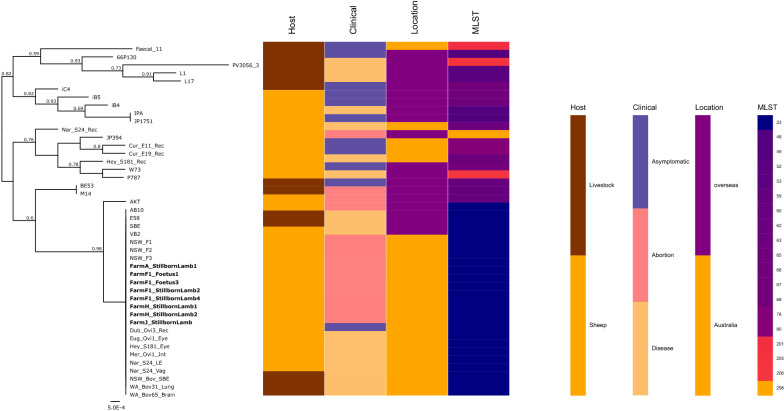
**Phylogenetic relationships of the *C. pecorum* strains from sheep and other livestock hosts.** The mid-point rooted tree was constructed using a 3095 bp concatenated MLST sequences alignment from the 44 *C. pecorum* strains, including the abortigenic *C. pecorum* strains described in this study (outlined in bold). Support values are displayed on the tree nodes. As displayed on the legend, metadata for each strain denotes: hosts including sheep and other livestock (goat, cattle and pig); clinical manifestations such as asymptomatic, abortion, and disease including Sporadic Bovine Encephalomyelitis (SBE), polyarthritis, pneumonia, orchitis, conjunctivitis, and metritis; geographical location (overseas and/or Australia) and STs. The figure displaying metadata was created with Phandango [69].

The *omp*A sequences from this study were analysed by BLASTn [50] to evaluate their % sequence similarity to Top BLAST hits, and aligned using ClustalOmega (as implemented in Geneious) to other publicly available *C. pecorum omp*A sequences retrieved from GenBank [51]. Using the 980 bp *omp*A alignment for the eight *C. pecorum* strains described in this study and additional 20 previously described strains, we constructed a mid-point rooted Bayesian phylogenetic tree, using MrBayes [52] as implemented in GeneiousPrime (Figure 1). The tree parameters included: GTR + I + G nucleotide substitution model, with four Markov Chain Monte Carlo chains of million generations, subsampled every 10 000 runs, and 100 000 trees discarded. The *omp*A sequences from this study were deposited in Genbank under accession numbers MW273771-MW273778. The MLST sequences were deposited in the *Chlamydiales* PubMLST database [48].

### Statistical analyses

Lamb mortality (%) between birth and marking for single-born lambs and multiple-born lambs (twins and triplets) were compared using two-tailed z-test [53]. Only farms where lambs were tagged at birth (Flocks A, B, C, E, G, H, I, J) were included in calculation of mortality for single- and multiple-born lambs. The proportion of cases with *C. pecorum* detected for each ewe age category (ewe lambs and yearling ewes) were compared using two-tailed z-test [53].

## Results

### Lamb mortality

From a total of 1963 lambs born, 1395 individual lamb records (including birth type, dam pedigree and survival) were available. Lamb mortality for study flocks are outlined in Additional file 4. Overall, lamb mortality from birth to marking ranged 12.6–27.1% for Merino yearling flocks and 9.3–40.7% for non-Merino ewe lamb flocks. Mortality rate for multiple-born lambs (twins or triplets) was 12% higher than for single-born lambs (*P* ≤ 0.001). One aborted foetus was recovered from Flock F1, and sequential pregnancy ultrasounds identified 7% of ewes with evidence of pregnancy loss occurring between day 80 and 117 from the start of mating in that flock. No overt evidence of outbreak of abortion (“abortion storm”) or ewe illness was observed by the farmers in any of the flocks during the study.

### Necropsies and cause of death

A total of 298 lamb necropsies were performed, which represented 69.1% of lambs that died between birth and marking. Remaining cases without necropsy either were not recovered by the farmers or died after 72 h of age.

The cause of death assigned at necropsy are shown in Additional file 5. Cause of death was established for 76% (227/298) of cases. The starvation–mismosthering–exposure complex, dystocia, and stillbirths accounted for 96% (218/227) of cases where cause of death was identified. Predation and decomposition were reported for 26% of necropsies. Overall, abortion, prematurity and stillbirth represented 21% necropsies where a cause of death category was assigned. Ewe death during the lambing period (*n* = 16) and subsequent death of their progeny was associated with 5% (23/431) lamb mortalities from birth to marking.

### Laboratory investigation for abortion and stillbirth cases—pathogen detection

Specimens for 35 cases classified as abortion or stillbirth from six farms were tested for evidence of infectious disease (Table 3 and Additional file 2). *Chlamydia pecorum* DNA was detected by qPCR in 39% (13/33) of stillborn or premature cases and 100% (2/2) of abortion cases, with *C. pecorum* detected at five of the six farms (Table 3)*. Chlamydia pecorum* DNA detection in aborted or stillborn progeny was higher for cases born to ewe lambs (64%, 14/22) compared with yearling Merino ewes (8%, 1/13; *P* = 0.001).

**Table 3:** **Detection of infectious agents from aborted or stillborn lambs in Western Australia**

	Yearlings		Ewe lambs					
	Flock A	Flock B	Flock F1	Flock F2	Flock H	Flock I	Flock J	Total
**Cases submitted** (*n*)								
Total	10	3	10	4	4	2	2	35
Aborted foetus & membranes	0	0	1	0	0	0	0	1
Aborted membranes only	0	0	1	0	0	0	0	1
Stillborn lamb	10	3	8	4	4	2	2^a^	33
***Chlamydia spp.***								
*C. pecorum*								
qPCR positive	1	0	9	0	3	1	1	15
Sequencing—*C. pecorum*	1	0	4	0	3	1	1	10
Insufficient amplification	0	0	5	0	0	0	0	5
*C. abortus*	0	0	0	0	0	0	0	0
*C. psittaci*	0	0	0	0	0	0	0	0
**Other**								
*Listeria* (culture)	0	0	0	0	0	0	0	0
*Salmonella* (culture)	0	0	0	0	0	0	0	0
*Trueperella pyogenes* (culture)	–	–	1	–	1	–	–	2
*Campylobacter* (culture)	0	0	0	0	0	0	0	0
*Campylobacter* (PCR)	0	0	2^b^	0	0	0	0	2^a^
*Leptospira* (PCR)	0	0	0	0	0	0	0	0
*Toxoplasma* (qPCR)	0	0	0	0	0	0	0	0
*Coxiella* (qPCR)	0	0	0	0	0	0	0	0
*Brucella* (PCR)	0	0	0	0	0	0	0	0
Pan-pestivirus (qPCR)	0	0	0	0	0	0	0	0

^a^Premature twins.

^b^*C. sputorum* and *C. mucosalis* by sequencing (suspected contaminant).

The only abortigenic bacteria isolated via culture was *Trueperella pyogenes* (*n* = 2), including in one case where *C. pecorum* was concurrently detected using qPCR. *Toxoplasma gondii, Listeria* spp., *Campylobacter fetus*, *Campylobacter jejuni* and exotic abortigenic agents (*C. abortus, B. melitensis, S. enterica* serovar Abortusovis) were not detected by culture or molecular diagnostics (Table 3).

### Laboratory investigation for abortion and stillbirth cases—histopathology

The majority of tissues submitted had significant autolysis and/or had been frozen which negatively impacted histopathological assessment (Additional file 6). In addition, due to the opportunistic nature of some of the sample collection, the range of tissues available for examination was variable, which prevented standardised evaluation of every individual. Cases where fixed tissues were available for histological assessment (*n* = 17) are summarised in Additional file 6. For cases in which *C. pecorum* was detected by qPCR and fixed tissue were available for histopathology (*n* = 9), lesions observed included placentitis (*n* = 4) epicarditis (*n* = 3), meningitis and encephalitis (*n* = 2), portal hepatitis and renal pyelitis (*n* = 2).

Necrotising placentitis with neutrophilic vasculitis was present for three lambs, with variable placental mineralisation. Another lamb displayed histiocytic infiltration of the allantoic mesenchyme without necrosis or vasculitis. Three of the five placental samples were subjected to *Chlamydia* IHC and all three were positive, with cytoplasmic staining of trophoblasts and macrophages.

Epicarditis was noted in three lambs, with mild, multifocal histiocytic and lymphocytic infiltrates present in each. Two lambs displayed a multifocal, histiocytic meningitis, moderate in intensity in one and mild in the second. The more severe case also had multifocal neutrophilic encephalitis and multifocal glial nodule formation. These sections were both *Chlamydia* spp. and *Toxoplasma gondii* IHC negative. Two lambs had mild, multifocal infiltrates of histiocytes and lymphocytes in the portal triads of the liver. Two lambs had histiocytic and variably neutrophilic infiltrates in the submucosa of the renal pelvis. The more severe case also displayed several renal epithelial cells distended by small, round, basophilic intracytoplasmic bodies compatible with chlamydial inclusions. The epithelium in the second case had sloughed and was unavailable for examination.

In addition, five lambs displayed meconium or squames in small pulmonary airways and five had a mild macrophage or neutrophil infiltrate in the alveoli. Of the five with inflammatory infiltrates in alveoli, two displayed positive staining of macrophage cytoplasm by *Chlamydia* IHC.

### Molecular characterisation of *C. pecorum* using MLST and ompA genotyping

The *C. pecorum* MLST and *omp*A genotyping was applied to samples from aborted (*n* = 2) or stillborn lambs (*n* = 6) from four farms, and compared to previously reported *C. pecorum* MLST and *omp*A sequences. The *C. pecorum* strain sequence types (STs) detected in the aborted and stillborn lambs were denoted as ST23. This genotype was identical to, and clustering with strains previously associated with pathogenicity including recently described ovine abortion from NSW (e.g. NSW_F1, NSW_F2, NSW_F3), ovine polyarthritis and/or conjunctivitis (e.g. Australian Mer_Ovi1_Jnt and Nar_S24_LE), and sporadic bovine encephalomyelitis (US E58, and Australian NSW/Bov/SBE, WA_Bov65_Brain) (Figure 1). The remaining *C. pecorum* STs that have been previously described are mainly sheep and cattle rectal strains, and strains from pig and goat hosts. These other ST clustered in three distinct and diverse larger clades.

Similarly, the *omp*A sequences detected in aborted and stillborn lambs in this study were genetically identical to previously reported isolates and strains associated with pathogenicity (e.g. Australian Mer/Ovi1/Jnt), US E58, and Australian NSW/Bov/SBE) clustering together in a well-supported clade (Additional file 7). The remaining the *omp*A sequences from sheep, goat and/or bovine rectal and other strains clustered in several genetically diverse clades (Additional file 7).

## Discussion

The detection of *C. pecorum* in aborted and stillborn lambs from primiparous ewes from multiple farms was the most striking observation in this study. *Chlamydia pecorum* has predominantly been associated with polyarthritis [54–56], keratoconjunctivitis [43] and asymptomatic gastrointestinal carriage and faecal shedding in Australian sheep [57]. Abortion due to *C. pecorum* is sporadic and not commonly reported [12, 18, 20], and the role of *C. pecorum* as an abortigenic agent is not well defined. Thereby, detection of this organism in aborted and stillborn lambs from multiple farms with no epidemiological or geographical relationship is notable, and *C. pecorum* should be considered as a differential diagnosis for abortion and perinatal mortality in Australian sheep.

Determining the aetiology in abortion and perinatal lamb death investigations is inherently challenging [12], and conclusive diagnosis of disease cannot be made based only on detection of a pathogen in tissue samples. Nevertheless, several observations from this study suggest *C. pecorum* was a likely aetiological agent associated with abortion and stillbirth on these farms. Firstly, other endemic and exotic abortigenic agents were not detected. Secondly, histopathological changes for cases where *C. pecorum* was detected were consistent with those reported for *C. abortus* and previously described *C. pecorum* abortion in small ruminants [18, 58, 59]. The high loads of *C. pecorum* detected in placenta and foetal liver from aborted and stillborn lambs (Table 2) was consistent with observations for other clinical diseases associated with *C. pecorum* [44, 55, 56]. Finally, MLST and *ompA* characterisation of high load *C. pecorum* DNA from aborted and stillborn lambs identified ST23 type strains that were identical to other globally distributed ST23 strains associated with pathology in sheep and cattle, including abortion [18, 60], arthritis [61, 62] and conjunctivitis [61, 62] in sheep, and sporadic bovine encephalopathy in cattle [44, 61]. Emerging evidence of abortigenic potential of *C. pecorum* is perhaps not surprising given the closely related *C. abortus* is an important cause of abortion in sheep in other countries, and *C. psittaci* is a cause of abortion in horses [63, 64].

*Chlamydia pecorum* was detected in aborted and stillborn lambs from five out of six farms. However, the degree to which *C. pecorum* ST23 contributed overall lamb mortalities could not be determined. Infectious disease screening was not conducted for lambs that died from causes other than abortion or stillbirth, including those classified as starvation-mismothering. However, similar to other bacterial infections of the pregnant uterus, it is likely that *C. pecorum*-associated placentitis results in a spectrum of outcomes, including abortion, stillbirths, lambs that are born alive, but weak and with low birth weights and poor survival, congenital infections or even normal offspring, depending on the severity of placental pathology and colonisation [18, 65]. Future investigations should determine whether infection contributes to reduced lamb viability, as well as abortion or stillbirth, and factors that impact outcome for infection.

*Chlamydia pecorum* detection was higher for aborted and stillborn progeny of younger ewes (ewe lambs) compared to yearling ewes. This was consistent with a recent case report from New South Wales, Australia where *C. pecorum* abortion was reported in primiparous ewe lambs, with no evidence of abortion storm in multiparous ewes on the same property [18]. Ewes mated as ewe lambs (under 12 months of age) may be more susceptible to *C. pecorum* ST23 infection and pathology. The reproductive performance of ewe lambs is highly variable. Improved understanding about the impact of *C. pecorum* ST23 on reproductive performance of ewe lambs and opportunities to mitigate impacts could inform management recommendations to improve their reproductive performance.

*Chlamydia pecorum* is endemic in Australian livestock [55, 57, 61], including sheep and cattle, and ubiquitous in livestock worldwide [64]. The route by which ewes became infected was not tested in our study. Faecal-oral transmission has been hypothesised, however mucosal shedding has been reported and transmission routes such as oculo- oral- or nasal contact, sexual transmission or inhalation are plausible [64].

Asymptomatic *C. pecorum* infections are commonly detected in sheep, with faecal carriage detected 30% faecal samples over three time points and flock point prevalence ranging 0–94% in Australian sheep [57]. However, MLST and *omp*A genotyping has demonstrated ST23 detected in cases of abortion, arthritis and conjunctivitis are distinct from gastrointestinal strains detected in rectal swabs (Figure 1). Therefore, studies that do not characterise *C. pecorum* ST cannot assess prevalence for the pathogenic ST23 genotype and the epidemiology for *C. pecorum* ST23 in Australian sheep remains poorly understood. Incidence of abortion, conjunctivitis and lameness are typically not reported for flocks included epidemiological studies because these conditions are challenging to detect in extensively managed sheep and may not be evident at the time of sampling. The incidence of conjunctivitis and arthritis was not able to be determined for flocks in our study, however polyarthritis associated with *C. pecorum* was detected in sheep from Farm F [56].

Overall lamb mortality for flocks in this study was comparable with ranges reported in other Australian studies [1]. Stillbirths accounted for 19% of necropsies where cause of death was determined, and despite the detection of *C. pecorum*, was not markedly different to stillbirths as proportion of total losses reported in other Australian studies [2]. Notably, stillbirths and abortion associated with *C. pecorum* ST23 were detected in flocks without overt evidence of abortion storm (i.e*.* observation of abortions by the farmer) or illness in ewes that would have normally triggered a veterinary investigation. This suggests *C. pecorum* may be associated with subclinical losses that go undetected on Australian farms, and explains why *C. pecorum* abortion is not more widely reported.

Listeriosis, campylobacteriosis and toxoplasmosis are the most common infectious causes of abortion and perinatal death in Australian sheep [12, 13]. These diseases are sporadic and were not detected in any of the aborted or stillborn lambs in this study. There was no evidence of exotic infectious diseases. *Trueperella pyogenes* was cultured in two cases, but the significance of this finding was not clear. Although *T. pyogenes* has been reported as a primary abortigenic agent [12, 66], the commensal nature of the organism on the mucosal surfaces predisposes aborted and birth material to secondary contamination [67]. Regarding the case where both *T. pyogenes* and *C. pecorum* were detected in the same lamb, co-infections with other pathogens has been reported for both species [31, 67]. However, the role of a synergistic interaction between *C. pecorum* and *T. pyogenes*, precipitating in disease, has not been established. This observation also serves as a reminder to consider mixed and co-infections during abortion investigations.

Starvation–mismosthering–exposure complex and dystocia accounted for most lamb mortalities that occurred in the perinatal period and mortality was higher for multiple-born lambs compared to single-born lambs. This was consistent with studies reported for multiparous ewes in Australia [1, 2, 5, 68], and primiparous ewes in New Zealand [9, 10]. Strategies to reduce dystocia and starvation–mismosthering–exposure, including provision of adequate shelter for lambing ewes and managing ewe nutrition during pregnancy to optimise lamb birthweights may help optimise survival for progeny of primiparous ewes [4].

The ability to assess brain and neurological tissue at necropsy was impacted by freezing of some carcasses and the extent of time lapsed prior to necropsy. This impacted comparison of proportion of mortalities attributable to dystocia relative to other studies that use brain and spinal cord lesion scores to determine cases as dystocia B (stillbirth) or dystocia C (birth injury) where obvious subcutaneous oedema of the head or neck is not present [2, 25]. Additionally, predation and decomposition were evident in approximately one quarter of necropsies, contributing to number of cases where cause of death could not be determined.

*Chlamydia pecorum* was detected in abortions and stillborn progeny of primiparous ewes from multiple farms and should be considered as a differential diagnosis for abortion and perinatal mortality in Australian sheep. The *C. pecorum* strains detected from abortions and stillborn lambs belong to the ST23 clade that has previously been associated with abortions in sheep and cattle, and other diseases including polyarthritis, conjunctivitis and sporadic bovine encephalitis. Further investigation to quantify impact of *C. pecorum* as a cause of abortion, stillbirth or poor lamb viability in sheep, and determine factors that impact infection outcome are warranted. Starvation–mismosthering–exposure complex, dystocia and stillbirths accounted for most lamb mortalities for lambs born to primiparous ewes, which is consistent with that reported for adult ewes.

## Supplementary Information

**Additional file 1.** **Cause of death classifications.**

**Additional file 2.** **Summary data for tissues available for laboratory diagnosis from aborted and stillborn lambs.**

**Additional file 3.** **Primer/probe final concentrations, sequences and cycling conditions for diagnostic PCRs performed during this study.**

**Additional file 4.** **Lamb mortality (birth to marking) for lambs born to primiparous ewes in Western Australia.**

**Additional file 5.** **Cause of lamb death identified at necropsy.**

**Additional file 6.** **Histopathology findings.**

**Additional file 7.** **The livestock *C. pecorum omp*A phylogenetic relationships.**

## Abbreviations

BLAST: Basic local alignment search tool
H&E: Haematoxylin and eosin
IHC: Immunohistochemistry
MLST: Multilocus sequence typing
ompA: Outer membrane protein A
PCR: Polymerase chain reaction
qPCR: Quantitative polymerase chain reaction
SME: Starvation–mismosthering–exposure complex
ST: Sequence type
