# Supplementary material for: Chlamydia pecorum detection in aborted and stillborn lambs from Western Australia
Source: Vet Res. 2021 Jun 11;52:84. doi: 10.1186/s13567-021-00950-w (PMC8196467; doi:10.1186/s13567-021-00950-w)
Supplement: Supplementary file 1 — Additional file 1. Cause of death classifications. [file 13567_2021_950_MOESM1_ESM.docx]

Classification Post-mortem observations

Dystocia Evidence of oedema to the head or neck

Stillborn Full-term appearance; not walked or breathed

Abortion/prematurity Pre-term appearance (size, wool covering); not walked or breathed

Starvation-mismothering-exposure complex Evidence that had walked and breathed

Empty stomach contents

Mobilisation of peri-renal and peri-cardial fat

Other Cause of death determined based on the gross

appearance of affected organ systems
