# Supplementary material for: Chlamydia pecorum detection in aborted and stillborn lambs from Western Australia
Source: Vet Res. 2021 Jun 11;52:84. doi: 10.1186/s13567-021-00950-w (PMC8196467; doi:10.1186/s13567-021-00950-w)
Supplement: Supplementary file 2 — Additional file 2. Summary data for tissues available for laboratory diagnosis from aborted and stillborn lambs. [file 13567_2021_950_MOESM2_ESM.docx]

| **Flock code** | **Report case number** | **Case code** | **Tissue submitted** | **Necropsy interpretation** |
| --- | --- | --- | --- | --- |
| A | AS-18-2738 | 18-001 | Brain, lung, liver | Stillborn |
| A | AS-18-2738 | 18-003 | Lung, placenta, liver | Stillborn |
| A | AS-18-2738 | 18-006 | Liver | Stillborn |
| A | AS-18-2738 | 18-008 | Liver, placenta | Stillborn |
| A | AS-18-2738 | 18-011 | Liver, placenta | Stillborn |
| A | AS-18-2738 | 18-013 | LiverC | Stillborn |
| A | AS-18-2960 | 18-024 | Liver | Stillborn |
| A | AS-18-2960 | 18-026 | Liver | Stillborn |
| A | AS-18-2960 | 18-028 | Liver | Stillborn |
| A | AS-18-2960 | 18-029 | LiverAB | Stillborn |
| B | AS-18-2882 | 18-040 | Liver, placenta, brain, lung, heart | Stillborn |
| B | AS-18-2882 | 18-050 | Liver, brain, placenta | Stillborn |
| B | AS-18-2882 | 18-051 | Liver, brain, placenta | Stillborn |
| F1 | AS-18-2650 | 18-079 | LiverAB, placenta, brain, stomach contents, | Abortion |
| F1 | AS-18-2736 | 18-080 | lung, kidney, heart PlacentaAB | Abortion |
| F1 | AS-18-2881 | 18-081 | LiverA | Stillborn |
| F1 | AS-18-2881 | 18-082 | LiverA | Stillborn |
| F1 | AS-18-2881 | 18-083 | LiverA, placentaAB, stomach contents | Stillborn |
| F1 | AS-18-2881 | 18-084 | Liver, placentaA | Stillborn |
| F1 | AS-18-2881 | 18-085 | Heart, lungA | Stillborn |
| F1 | AS-18-2961 | 18-087 | LiverAB, placenta | Stillborn |
| F1 | AS-18-2961 | 18-089 | Brain, placenta | Stillborn |
| F1 | AS-18-2961 | 18-091 | LiverAB, brain, lung | Stillborn |
| F2 | AS-19-3155 | 19-111 | Liver, lung, placenta | Stillborn |
| F2 | AS-19-3155 | 19-112 | Liver, lung, placenta | Stillborn |
| F2 | AS-19-3155 | 19-114 | Liver, lung, placenta | Stillborn |
| F2 | AS-19-3155 | 19-160 | Liver, lung, brain, heart, kidney | Stillborn |
| H | AS-19-2600 | 19-002 | Liver, stomach, placentaA, lung | Stillborn |
| H | AS-19-2600 | 19-003 | Liver, lung | Stillborn |
| H | AS-19-2600 | 19-028 | LiverAC, placentaAB, lung, stomach contents | Stillborn |
| H | AS-19-2600 | 19-030 | LiverAB, placentaA, lung | Stillborn |
| I | AS-19-2758 | 19-032 | Liver, lung | Stillborn |
| I | AS-19-2758 | 19-042 | Liver, kidney, heart, lung | Stillborn |
| J | AS-19-2601 | 19-007 | LiverAB, lung, brain, clotted heart blood, | Premature |
| J | AS-19-2601 | 19-008 | stomach content, heart, kidney  LiverA, lung, brain, clotted heart blood, | Premature |
|  |  |  | stomach content, heart, kidney |  |

A Tissue samples in which *C. pecorum* was detected using qPCR

B Tissue samples from which *C. pecorum* was characterised (MLST and *omp*A)

C Tissue samples where *T. pyogenes* was cultured
