# Supplementary material for: Chlamydia pecorum detection in aborted and stillborn lambs from Western Australia
Source: Vet Res. 2021 Jun 11;52:84. doi: 10.1186/s13567-021-00950-w (PMC8196467; doi:10.1186/s13567-021-00950-w)
Supplement: Supplementary file 3 — Additional file 3. Primer/probe final concentrations, sequences and cycling conditions for diagnostic PCRs performed during this study. [file 13567_2021_950_MOESM3_ESM.docx]

**Additional File 3**: Primer/probe final concentrations, sequences and cycling conditions for diagnostic PCRs performed during this study A

| **Assay** | **Primer/probe sequences and final PCR conc.** | **Cycling conditions** | **References** |
| --- | --- | --- | --- |
| *Brucella* spp. | ISP1 - GGT TGT TAA AGG AGA (1.0 uM) | Hold: 95 °C for 5 min | [35] |
| PCR | ISP2 - GAC GAT AGC GTT TCA (1.0 uM) | 35 cycles: 94 °C for 30 s, |  |
|  |  | 55 °C for 30 s, |  |
|  |  | 72 °C for 1 min |  |
|  |  | Hold: 72 °C for 6 min |  |
| *Campylobacter* | C412F - GGA TGA CAC TTT TCG GAG C (0.4 uM) | Hold: 95 °C for 5 min | [36] |
| spp. PCR | C1288R - CAT TGT AGC ACG TGT GTC (0.4 uM) | 30 cycles: 94 °C for 30 s, |  |
|  |  | 54 °C for 30 s, |  |
|  |  | 72 °C for 1 min |  |
|  |  | Hold: 72 °C for 7 min |  |
| *Chlamydia* | CpaOMP1-F - GCA ACT GAC ACT AAG TCG GCT ACA (0.5 uM) | Hold: 95 °C for 5 min | [32] |
| *abortus* qPCR | CpaOMP1-R - ACA AGC ATG TTC AAT CGA TAA GAG A (0.5 uM) | 40 cycles: 95 °C for 15 s, |  |
|  | CpaOMP1-S - FAM-TAA ATA CCA CGA ATG GCA AGT TGG TTT AGC G-BHQ-1 (0.2 uM) | 60 °C for 15 s (acquiring green) |  |
| *Chlamydia* | CppecOMP1-F - CCA TGT GAT CCT TGC GCT ACT (0.5 uM) | Hold: 95 °C for 5 min | [31] |
| *pecorum* qPCR | CppecOMP1-R - TGT CGA AAA CAT AAT CTC CGT AAA AT (0.5 uM) | 40 cycles: 95 °C for 15 s, |  |
|  | CppecOMP1-S - FAM-TGC GAC GCG ATT AGC TTA CGC GTA G-BHQ-1 (0.2 uM) | 60 °C for 15 s (acquiring green) |  |
| *Chlamydia* | CppsOMP1-F - CAC TAT GTG GGA AGG TGC TTC A (0.5 uM) | Hold: 95 °C for 5 min | [32] |
| *psittaci* qPCR | CppsOMP1-R - CTG CGC GGA TGC TAA TGG (0.5 uM) | 40 cycles: 95 °C for 15 s, |  |
|  | CppsOMP1-S - FAM-CGC TAC TTG GTG TGA C-BHQ-plus (0.2 uM) | 60 °C for 15 s (acquiring green) |  |
| *Chlamydiales* | 16SIGF - CGG CGT GGA TGA GGC AT (0.4 uM) | Hold: 95 °C for 5 min | [33] |
| PCR (1) | 16SIGR - TCA GTC CCA GTG TTG GC (0.4 uM) | 40 cycles: 94 °C for 30 s, |  |
|  |  | 55 °C for 45 s, |  |
|  |  | 72 °C for 45 s |  |
|  |  | Hold: 72 °C for 7 min |  |
| *Chlamydiales* | 16SIGF - CGG CGT GGA TGA GGC AT (0.4 uM) | Hold: 95 °C for 5 min | [33] |
| PCR (2) | 806R - GGA CTA CCA GGG TAT CTA AT (0.4 uM) | 40 cycles: 94oC for 30 sec, |  |
|  |  | 55 °C for 45 sec, |  |
|  |  | 72 °C for 45 sec |  |
|  |  | Hold: 72 °C for 7 min |  |

| **Assay** | **Primer/probe sequences and final PCR conc.** | **Cycling conditions** | **References** |
| --- | --- | --- | --- |
| *Coxiella burnetii* | com1F - AAA ACC TCC GCG TTG TCT TCA (0.4 uM) | Hold: 50 °C for 3 min | [38] |
| multiplex qPCR | com1R - GCT AAT GAT ACT TTG GCA GCG TAT TG (0.4 uM) | Hold: 95 °C for 5 min |  |
|  | com1probe - FAM-AGA ACT GCC CAT TTT TGG CGG CCA-BHQ-1 (0.2 uM) | 60 cycles: 95 °C for 20 s, |  |
|  |  | 60 °C for 40 s (acquiring green/yellow) |  |
|  | IS1111aF - GTT TCA TCC GCG GTG TTA AT (0.2 uM) |  | [37] |
|  | IS1111aR - TGC AAG AAT ACG GAC TCA CG (0.2 uM) |  |  |
|  | IS1111aP - TET-CCC ACC GCT TCG CTC GCT AA-BHQ-1 (0.1 uM) |  |  |
| Pathogenic | Lepto F - CCC GCG TCC GAT TAG (0.5 uM) | Hold: 95 °C for 5 min | [39] |
| *Leptospira* spp. | Lepto R - TCC ATT GTG GCC GRA CAC (0.5 uM) | 40 cycles: 95 °C for 15 s, |  |
| qPCR | Lepto P - FAM-CTC ACC AAG GCG ACG ATC GGT AGC-TAMRA (0.2 uM) | 60 °C for 15 s (acquiring green) |  |
| Pan-Pestivirus | Pesti-3F - CCT GAG TAC AGG RTA GTC GTC A (0.9 uM) | Hold: 45 °C for 10 min | [40] |
| RT-qPCRB | Pesti-4R - GGC CTC TGC AGC ACC CTA TCA (0.9 uM) | Hold: 95 °C for 10 min |  |
|  |  | 45 cycles: 95 °C for 15 s, |  |
|  |  | 60 °C for 45 s (acquiring green) |  |
|  | BVDV190 - GRA GTC GTC ART GGT TCG AC (0.9 uM) |  | [41] |
|  | V326 - TCA ACT CCA TGT GCC ATG TAC (0.9 uM) |  |  |
|  | TQ-pesti-P - FAM-TGC YAY GTG GAC GAG GGC ATG C-BHQ-1 (0.25 uM) |  |  |

^A^ All qPCRs used Rotor-Gene Multiplex Master Mix (Qiagen) and were run on a Rotor-Gene Q (Qiagen) real-time PCR cycler except where noted. All conventional PCRs used HotStarTaq Master Mix (Quagen) and were performed on a DNA Engine (Bio-Rad) thermal cycler.

^B^ AgPath-ID One-Step RT-PCR Master Mix (Applied Biosystems) was used for the Pan-Pestivirus RT-qPCR and performed on an Applied Biosystems 7500 Real-Time PCR instrument.
