# Supplementary material for: Chlamydia pecorum detection in aborted and stillborn lambs from Western Australia
Source: Vet Res. 2021 Jun 11;52:84. doi: 10.1186/s13567-021-00950-w (PMC8196467; doi:10.1186/s13567-021-00950-w)
Supplement: Supplementary file 4 — Additional file 4. Lamb mortality (birth to marking) for lambs born to primiparous ewes in Western Australia. [file 13567_2021_950_MOESM4_ESM.docx]

|  | | Mortality (%)^A^ |  | |
| --- | --- | --- | --- | --- |
| Flock code | Lambs born (*n*) | All birth types | Singles | Multiple |
| A | 249 | 19.7 | 9.8 | 29.4 |
| B | 210 | 27.1 | 17.2 | 42.7 |
| C | 277 | 23.5 | 16.0 | 29.1 |
| D | 151 ^B^ | 12.6 ^B^ | Unknown | Unknown |
| E | 89 | 18.0 | 7.0 | 25.0 |
| F1 | 169 ^B^ | 29.0 ^B^ | Unknown | Unknown |
| F2 | 150 ^B^ | 40.7 ^B^ | Unknown | Unknown |
| G | 197 | 16.8 | 15.4 | 17.2 |
| H | 196^C^ | 10.7 ^C^ | 15.8 ^C^ | 14.5 ^C^ |
| I | 145 | 20.0 | 15.7 | 24.2 |
| J | 130 | 24.6 | 13.0 | 29.2 |
| TOTAL | 1963 | 22.0 | 14.0 ^D^ | 26.4 ^D^ |

^A^ Birth to marking

^B^ Lambs not tagged at birth (number lambs born may be underestimated)

^C^ Not all lambs tagged and assigned to birth type and/or dam

^D^ Mortality calculated based on data only from flocks that had individual lamb survival data (birth type, rear type, dam pedigree)
