# Supplementary material for: Chlamydia pecorum detection in aborted and stillborn lambs from Western Australia
Source: Vet Res. 2021 Jun 11;52:84. doi: 10.1186/s13567-021-00950-w (PMC8196467; doi:10.1186/s13567-021-00950-w)
Supplement: Supplementary file 5 — Additional file 5. Cause of lamb death identified at necropsy. [file 13567_2021_950_MOESM5_ESM.docx]

Cause of death category (*n*)

| Flock code | **Necropsies**  *n* (%)^A^ | Abortion &  premature | Stillbirth | Dystocia | SMEB^B^ | Infection | Undetermined |
| --- | --- | --- | --- | --- | --- | --- | --- |
| A | 34 (69.4) | 0 | 11 | 8 | 7 | 0 | 8 |
| B | 39 (68.4) | 0 | 3 | 5 | 16 | 0 | 15 |
| C | 32 (49.2) | 0 | 3 | 3 | 16 | 3 | 7 |
| D | 19 (100)^C^ | 0 | 1 | 0 | 13 | 1 | 4 |
| E | 7 (43.8) | 0 | 2 | 3 | 0 | 0 | 2 |
| F1 | 49 (100) ^C^ | 2 | 11 | 13 | 10 | 0 | 13 |
| F2 | 48 (78.7) ^C^ | 0 | 3 | 14 | 23 | 1 | 7 |
| G | 12 (36.4) | 0 | 1 | 7 | 2 | 0 | 2 |
| H | 21 (100) ^C^ | 0 | 4 | 3 | 7 | 0 | 7 |
| I | 23 (79.3) | 0 | 3 | 15 | 2 | 0 | 3 |
| J | 14 (43.8) | 2 | 2 | 2 | 5 | 0 | 3 |
| Total (*n*) | 298 | 4 | 44 | 73 | 101 | 5 | 71 |
| Total (% necropsy) | - | 1 | 15 | 24 | 34 | 2 | 24 |
| **Total** (% necropsy with diagnosis) | - | 1.8 | 19.4 | 32.1 | 44.5 | 2.2 | - |
| **Total** (% lambs born) | 15.2 | - | - | - | - | - | - |

^A^ Lamb necropsies expressed as % of all lamb mortalities birth to marking

^B^ SME: Starvation-mismothering-exposure complex

^C^ Lambs not tagged at birth (necropsy proportion may be overestimated)
