# Supplementary material for: Chlamydia pecorum detection in aborted and stillborn lambs from Western Australia
Source: Vet Res. 2021 Jun 11;52:84. doi: 10.1186/s13567-021-00950-w (PMC8196467; doi:10.1186/s13567-021-00950-w)
Supplement: Supplementary file 6 — Additional file 6. Histopathology findings. [file 13567_2021_950_MOESM6_ESM.docx]

| **Flock** | **Cause of death** | **Report case number** | **Case code** | **Histopathology findings** |
| --- | --- | --- | --- | --- |
| A | Stillborn | AS-18-2738 | 18-001, liver | NSF |
|  |  |  | 18-001, lung | NSF |
|  |  |  | 18-001, brain | NSF |
| A | Stillborn | AS-18-2738 | 18-003, liver | NSF |
|  |  |  | 18-003, placenta | NSF |
|  |  |  | 18-003, lung | NSF |
| F1 | Abortion | AS-18-2650* | 18-079, liver | Multifocal *Chlamydia* IHC staining of Kupffer cells |
|  |  |  | 18-079, placenta  18-079, lung | Necrotising placentitis with neutrophilic vasculitis;  *Chlamydia* IHC positive  Multifocal *Chlamydia* IHC staining of alveolar macrophages |
|  |  |  | 18-079, heart  18-079, brain  18-079, kidney | Mild, multifocal histiocytic, lymphocytic & neutrophilic epicarditis  Mild, diffuse, neutrophilic, histiocytic & lymphocytic meningitis  Autolysed. Focal histiocytic & neutrophilic pyelitis |
| F1 | Abortion | AS-18-2736* | 18-080, placenta | Autolysed. Multifocal necrotising placentitis with |
| F1 | Stillborn | AS-18-2881* | 18-081, liver | mineralisation and multifocal *Chlamydia* IHC staining NSF |
| F1 | Stillborn | AS-18-2881* | 18-083, liver | NSF |
| F1 | Stillborn | AS-18-2881* | - 1. , placenta   2. , liver | Multifocal, necrotising placentitis with mineralisation and focal neutrophilic vasculitis  NSF |
| F1 | Stillborn | AS-18-2881* | - 1. , placenta   2. , lung | Mild, diffuse histiocytic placentitis with multifocal  *Chlamydia* IHC staining NSF |
|  |  |  | 18-085, heart | NSF |
| F1 | Stillborn | AS-18-2961* | 18-091, liver | Multifocal mild histiocytic portal hepatitis |
|  |  |  | 18-091, brain  18-091, lung | Moderate, multifocal neutrophilic encephalitis with mild histiocytic meningitis  Low numbers of neutrophils and macrophages in airways |
| F2 | Stillborn | AS-19-3155 | 19-111, liver | NSF |
|  |  |  | 19-111, placenta | NSF |
|  |  |  | 19-111, lung | Meconium in small airways |
| F2 | Stillborn | AS-19-3155 | 19-112, liver | NSF |
|  |  |  | 19-112, placenta | NSF |
|  |  |  | 19-112, lung | Meconium in small airways |
| F2 | Stillborn | AS-19-3155 | 19-114, liver | NSF |
|  |  |  | 19-114, placenta | NSF |
|  |  |  | 19-114, lung | Meconium in small airways |
| F2 | Stillborn | AS-19-3155 | 19-160, liver | Moderate, multifocal periacinar hepatic necrosis |
|  |  |  | 19-160, lung | Meconium in small airways |
|  |  |  | 19-160, heart | NSF |
|  |  |  | 19-160, brain | NSF |
| I | Stillborn | AS-19-2758 | 19-032, liver | NSF |
|  |  |  | 19-032, lung | NSF |

| **Flock** | **Cause of death** | **Report case number** | **Case code** | **Histopathology findings** |
| --- | --- | --- | --- | --- |
| I | Stillborn | AS-19-2758* | 19-042, liver | Mild, multifocal lymphocytic & histiocytic portal hepatitis |
|  |  |  | 19-042, lung | Mild, multifocal proliferation of alveolar macrophages |
|  |  |  | 19-042, heart | NSF |
|  |  |  | 19-042, kidney | NSF |
| J | Premature | AS-19-2601* | 19-007, liver | NSF |
|  |  |  | 19-007, lung  19-007, heart | Mild multifocal alveolar macrophage proliferation Multifocal, mild lymphocytic & histiocytic epicarditis & myocarditis |
|  |  |  | 19-007, brain  19-007, kidney | NSF  Diffuse, marked lymphocytic & histiocytic pyelitis with intraepithelial, intracytocplasmic inclusion bodies indicative of *Chlamydia* |
| J | Premature | AS-19-2601 | 19-008, liver  19-008, lung | NSF  Mild, multifocal alveolar macrophage proliferation with squames in small airways |
|  |  |  | 19-008, heart | NSF |
|  |  |  | 19-008, brain | NSF |
|  |  |  | 19-008, kidney | NSF |

NSF: no significant findings IHC: immunohistochemistry

* Cases in which *C. pecorum* was detected by qPCR
