# Supplementary material for: Chlamydia pecorum detection in aborted and stillborn lambs from Western Australia
Source: Vet Res. 2021 Jun 11;52:84. doi: 10.1186/s13567-021-00950-w (PMC8196467; doi:10.1186/s13567-021-00950-w)
Supplement: Supplementary file 7 — Additional file 7. The livestock C. pecorum ompA phylogenetic relationships. [file 13567_2021_950_MOESM7_ESM.pdf]

Additional File 7: The livestock *C. pecorum ompA* phylogenetic relationships [A]

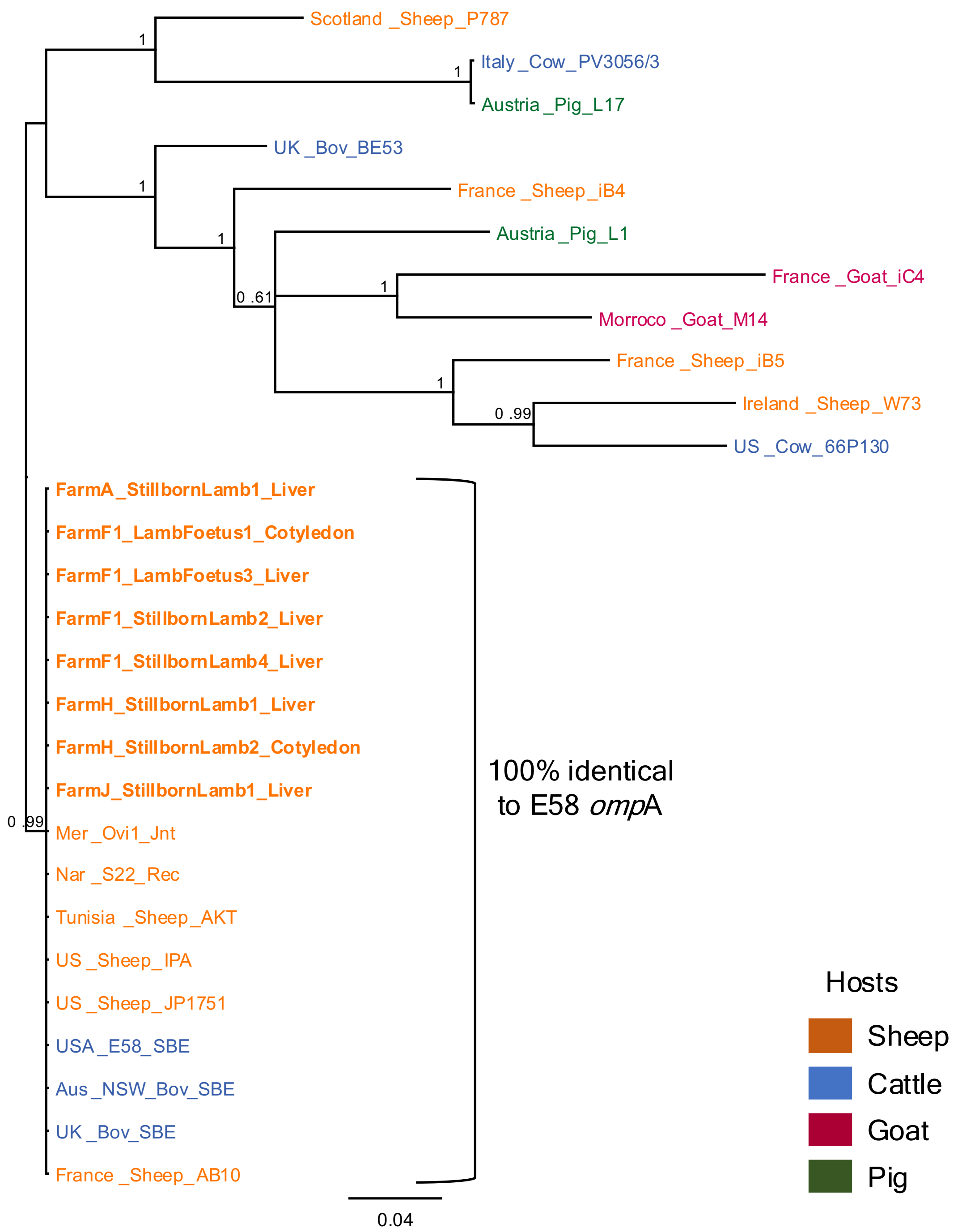

[A] The mid-point rooted Bayesian tree was constructed using the 980 bp *ompA* alignment sequences from 28 *C. pecorum* strains from livestock (sheep, goat, cattle and pig) hosts, including the lamb abortigenic *C. pecorum* strains described in this study. The lamb abortigenic strains are denoted in bold, whilst the hosts are coloured as in the legend. Posterior probabilities are displayed on the tree nodes.
